# Impact of velocity- and acceleration-compensated encodings on signal dropout and black-blood state in diffusion-weighted magnetic resonance liver imaging at clinical TEs

**DOI:** 10.1371/journal.pone.0291273

**Published:** 2023-10-05

**Authors:** Tobit Führes, Marc Saake, Filip Szczepankiewicz, Sebastian Bickelhaupt, Michael Uder, Frederik Bernd Laun

**Affiliations:** 1 Institute of Radiology, University Hospital Erlangen, Friedrich-Alexander-Universität Erlangen-Nürnberg (FAU), Erlangen, Germany; 2 Medical Radiation Physics, Clinical Sciences Lund, Lund University, Lund, Sweden; Feinstein Institute for Medical Research Fertility Research Laboratory: Northwell Health Feinstein Institutes for Medical Research, UNITED STATES

## Abstract

**Purpose:**

The study aims to develop easy-to-implement concomitant field-compensated gradient waveforms with varying velocity-weighting (*M*_1_) and acceleration-weighting (*M*_2_) levels and to evaluate their efficacy in correcting signal dropouts and preserving the black-blood state in liver diffusion-weighted imaging. Additionally, we seek to determine an optimal degree of compensation that minimizes signal dropouts while maintaining blood signal suppression.

**Methods:**

Numerically optimized gradient waveforms were adapted using a novel method that allows for the simultaneous tuning of *M*_1_- and *M*_2_-weighting by changing only one timing variable. Seven healthy volunteers underwent diffusion-weighted magnetic resonance imaging (DWI) with five diffusion encoding schemes (monopolar, velocity-compensated (*M*_1_ = 0), acceleration-compensated (*M*_1_ = *M*_2_ = 0), 84%-*M*_1_–*M*_2_-compensated, 67%-*M*_1_–*M*_2_-compensated) at b-values of 50 and 800 s/mm^2^ at a constant echo time of 70 ms. Signal dropout correction and apparent diffusion coefficients (ADCs) were quantified using regions of interest in the left and right liver lobe. The blood appearance was evaluated using two five-point Likert scales.

**Results:**

Signal dropout was more pronounced in the left lobe (19%-42% less signal than in the right lobe with monopolar scheme) and best corrected by acceleration-compensation (8%-10% less signal than in the right lobe). The black-blood state was best with monopolar encodings and decreased significantly (p < 0.001) with velocity- and/or acceleration-compensation. The partially *M*_1_–*M*_2_-compensated encoding schemes could restore the black-blood state again. Strongest ADC bias occurred for monopolar encodings (difference between left/right lobe of 0.41 μm^2^/ms for monopolar vs. < 0.12 μm^2^/ms for the other encodings).

**Conclusion:**

All of the diffusion encodings used in this study demonstrated suitability for routine DWI application. The results indicate that a perfect value for the level of *M*_1_–*M*_2_-compensation does not exist. However, among the examined encodings, the 84%-*M*_1_–*M*_2_-compensated encodings provided a suitable tradeoff.

## Introduction

Diffusion weighted magnetic resonance imaging (DWI) of the liver is widely used for several tasks, including detection of focal lesions [1–3], evaluation of treatment response of tumors [4, 5], and detection and classification of fatty liver diseases and liver fibrosis [6, 7].

However, a significant disadvantage of liver DWI is the frequent occurrence of signal dropout in regions close to the heart, caused by the bulk motion of the liver that induces dephasing and subsequent signal loss [8–11].

Previous studies have demonstrated that motion-compensated encodings, which are insensitive to motion at constant velocity and/or constant acceleration [12–17], are useful in correcting this signal loss [14, 18–20]. Compensation of the first gradient moment *M*_1_ (i.e. velocity-compensation) has been shown to reduce the signal loss considerably, yet not completely [10]. Post-processing schemes can ameliorate the problem [20], but a compensation at the sequence level is more desirable. The compensation of the second gradient moment *M*_2_ (acceleration-compensation) has thus been proposed. Application of motion compensation results in cancellation of dephasing caused by motion. However, blood vessels might appear bright [10] and confound lesion detection. Therefore, small velocity-weightings have been suggested instead of full motion-compensation [15, 21, 22].

Concerning the exact implementation of gradient-moment compensated diffusion encodings, Aliotta et al. [23] proposed the convex optimized diffusion encoding (CODE) optimization algorithm to create asymmetric echo time-efficient motion-compensated waveforms. They demonstrated a significant reduction in the echo time. However, asymmetric gradient waveforms may lead to non-balanced concomitant gradients [24, 25], which induce a spatially varying dephasing. This problem was addressed by Pena-Nogales et al. [16], who proposed a numerical optimization of waveforms that combined compensation for undesired effects from both motion encoding and concomitant gradients.

To complement these numerically optimized waveforms, we here propose maintaining a symmetric waveform design [26] that is easily described analytically. The symmetry of the waveform inherently compensates for concomitant gradient effects. In addition, it is feasible to implement them without the requirement for time-consuming optimization procedures.

Moreover, we took up the idea of incorporating small motion-weightings and propose a method to tune the level of velocity and acceleration-compensation towards arbitrary values by only changing one analytic parameter describing the waveform, which enabled a constant echo time for all waveforms.

While both signal dropout correction and insufficient blood suppression are related to motion compensation, their response to small motion-weightings can vary. To find a balance between the two, we aimed to find an optimal setting for *M*_1_ and *M*_2_. We thought that this "sweet spot" would allow us to achieve a black-blood state while simultaneously reducing the pulsation artifact.

The primary goal of this study is to evaluate analytically describable waveforms that are compensated for concomitant gradient effects for DWI of the liver. These waveforms possess the unique quality of controllable *M*_1_ and *M*_2_ weighting through a single parameter that changes the duration of the single gradient pulses while not changing the total encoding duration. We assessed their performance in volunteer measurements focusing on signal dropout correction and the visualization of blood appearance. We chose a constant echo time for all imaging experiments to achieve comparability and assessed whether there might be a point of compromise between the black-blood state and signal dropout correction.

## Methods

The study was approved by: Ethics committee of the Friedrich-Alexander-University Erlangen-Nürnberg.—Written informed consent was obtained.

### Imaging

Diffusion-weighted images of seven healthy volunteers (aged 23–28 years, male/female 3/4) with no background of liver disease were acquired on a 3T MAGNETOM Prisma scanner (Siemens Healthineers, Erlangen, Germany). Volunteers were recruited in January and February 2023. We used an in-house developed single-refocused diffusion echo-planar imaging (EPI) sequence [27, 28], which allows arbitrarily shaped diffusion encodings. Data were acquired during breath-hold in expiration and stored pseudonymously according to the ethics approval. The used imaging parameters are shown in Table 1.

**Table 1:** Sequence parameters.

Slices	11
Slice thickness	5 mm
Distance between slices	5 mm
FoV (read × phase)	350 mm × 357 mm
TR	2,500 ms
TE	70 ms
Fat saturation	SPAIR
Surface coil flare	Compensated with “prescan normalize” option
Matrix size	100 × 102 (interpolated to 200 × 204)
Phase partial Fourier factor	6/8
Bandwidth	2272 Hz/Px
Diffusion weightings (repetitions)	50 s/mm^2^ (1), 800 s/mm^2^ (5)
Diffusion directions	3 (orthogonal)

### Diffusion encodings

Diffusion encodings usually induce a phase accumulation for moving spins. Different movements induce different phase accumulations. Within a voxel, this means a loss of phase coherence, which induces a signal loss. This can be avoided by employing velocity compensation, which has the effect that spins moving with constant velocity accumulate no net phase. A diffusion encoding *G*(*t*) is velocity-compensated if its first gradient moment is zero:

$$M_{1}=\gamma \int_{0}^{TE}G(t)\cdot tdt=0,$$

with *γ* = 2.678 × 10^8^ rad/s/T. The phase accumulation can also be nulled for spins experiencing a constant acceleration. This is achieved by using diffusion encodings with zero second gradient moment:

$$M_{2}=\gamma \int_{0}^{TE}G(t)\cdot t^{2}dt=0.$$

**Fig 1:**
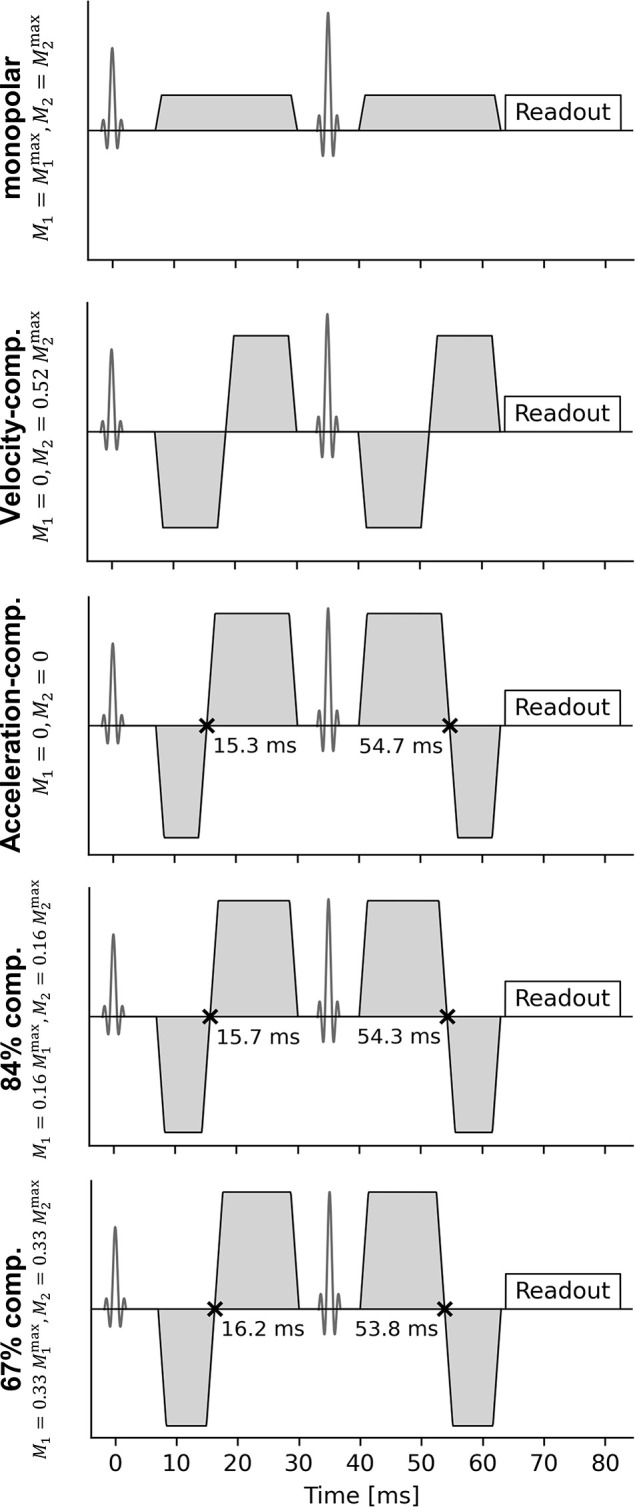
Used diffusion encodings. Note the slight difference in the duration of the single trapezoidal gradient pulses in row 3 to 5, which is necessary to achieve the different values of *M*_1_ and *M*_2_ weightings.

Five diffusion encoding schemes were used (Fig 1):

- Monopolar diffusion encoding ($M_{1}=M_{1}^{max},M_{2}=M_{2}^{max}$)
- Velocity-compensated diffusion encoding ($M_{1}=0,M_{2}=0.52\;M_{2}^{max}$)
- Acceleration-compensated diffusion encoding (*M*_1_ = 0, *M*_2_ = 0)
- 84%-compensated diffusion encoding ($M_{1}=0.16\;M_{1}^{max},M_{2}=0.16\;M_{2}^{max}$)
- 67%-compensated diffusion encoding ($M_{1}=0.33\;M_{1}^{max},M_{2}=0.33\;M_{2}^{max}$)

The absolute values of *M*_1_ and *M*_2_ are shown in Table 2.

**Table 2:** Absolute values of *M*_1_ and *M*_2_.

	*M*_1_[s/mm]		*M*_2_[s^2^/mm]	
	b = 50 s/mm^2^	b = 800 s/mm^2^	b = 50 s/mm^2^	b = 800 s/mm^2^
Monopolar	1.46	5.83	0.082	0.33
Velocity-compensated	0	0	0.042	0.17
Acceleration-compensated	0	0	0	0
84%-compensated	0.23	0.93	0.013	0.052
67%-compensated	0.49	1.96	0.027	0.11

To achieve the desired echo time of 70 ms, the duration of the diffusion encoding was set to 56 ms (time before 180° pulse: 23 ms, pause: 10 ms, time after 180° pulse: 23 ms, adding up to 56 ms), which allowed for enough time for the readout.

The gradient pulses of the monopolar and acceleration-compensated gradient waveforms were generated with the optimization toolbox NOW [14, 29, 30].

The gradient pulses for velocity-compensated encodings were created manually. For a given gradient pulse *G*_pulse_(*t*), a velocity-compensated encoding can be created from this pulse by concatenating *G*_pulse_(*t*), −*G*_pulse_(*t*), −*G*_pulse_(*t*), *G*_pulse_(*t*) in time domain, where a pause of arbitrary length can be included between the second and third element [26, 31]. Here, a single trapezoidal pulse with a duration of 11.5 ms (rise time of 1.3 ms) was used as *G*_pulse_(*t*). The pause in between was again set to 10 ms. The result is shown in Fig 1 (line 2, “velocity-comp.”). Note that the first part of the encoding has been flipped to account for the 180-degree pulse.

As an alternative to numerically optimized waveforms, the 84%- and 67%-compensated encodings were also created manually based on the acceleration-compensated encoding. The concept is first explained with idealized rectangular (instead of realistic trapezoidal) gradient pulses. The following variables are used:

- *a*: the duration of the short gradient pulses (cf. first and fourth pulse in Fig 1, line 3)
- *b*, the duration of the long gradient pulses (cf. second and third pulse)
- *c*, the pause length.

These parameters are also visualized in S1 File.

The *M*_2_ value of this scheme with rectangular pulses is proportional to:

$$M_{2}\propto -2a^{3}+2b^{3}-6a^{2}b-3a^{2}c+3b^{2}c-2ab^{2}-ac^{2}+bc^{2}-2abc.$$

By changing *a* to *a*+*t*_*S*_ and *b* to *b*−*t*_*S*_, while not changing *c*, *M*_1_(*t*_*S*_) and *M*_2_(*t*_*S*_) are in good approximation linear in *t*_*S*_:

$$M_{1}(t_{S})=\sqrt{3b_{diff}}\frac{-2(2b+c)\cdot t_{S}}{\sqrt{2a^{3}+3a^{2}(2b+c)-6ab(b+c)+b^{2}(2b+3c)}}+O({t_{S}}^{2})$$

$$M_{2}(t_{S})=\sqrt{3b_{diff}}\frac{-2(2b+c)(2a+2b+c)\cdot t_{S}}{\sqrt{2a^{3}+3a^{2}(2b+c)-6ab(b+c)+b^{2}(2b+3c)}}+O({t_{S}}^{2}).$$

The value *b*_diff_ denotes the b-value. The full calculations are shown in the Supporting Information.

For realistic trapezoidal gradient pulses, the linearity in *t*_*S*_ for small *t*_*S*_ (*t*_*S*_<1.5 ms) still holds. The values of *M*_1_ and *M*_2_ change by the same relative amount with increasing *t*_*S*_ (see S1 File). In our setting, this value can be approximated by:

$$\frac{M_{1}}{M_{1}^{max}}(t_{S})=\frac{M_{2}}{M_{2}^{max}}(t_{S})\approx 0.348\frac{t_{S}}{ms}.$$

$M_{1}^{max}$ and $M_{2}^{max}$ denote *M*_1_ and *M*_2_ of the monopolar gradient at the same b-value and the same pulse length and pause length (as shown in Fig 1). At b = 800 s/mm^2^, $M_{1}^{max}\approx 5.8\;s/mm$ and $M_{2}^{max}\approx 0.33\;s^{2}/mm$. We used values of *t*_*S*_ = 0.45 ms and *t*_*S*_ = 0.95 ms, leading to values of $\frac{M_{1}}{M_{1}^{max}}(t_{S})=\frac{M_{2}}{M_{2}^{max}}(t_{S})$ of 0.84 and 0.67.

### Evaluation

All calculations were performed using Python 3.7.9.

**Fig 2:**
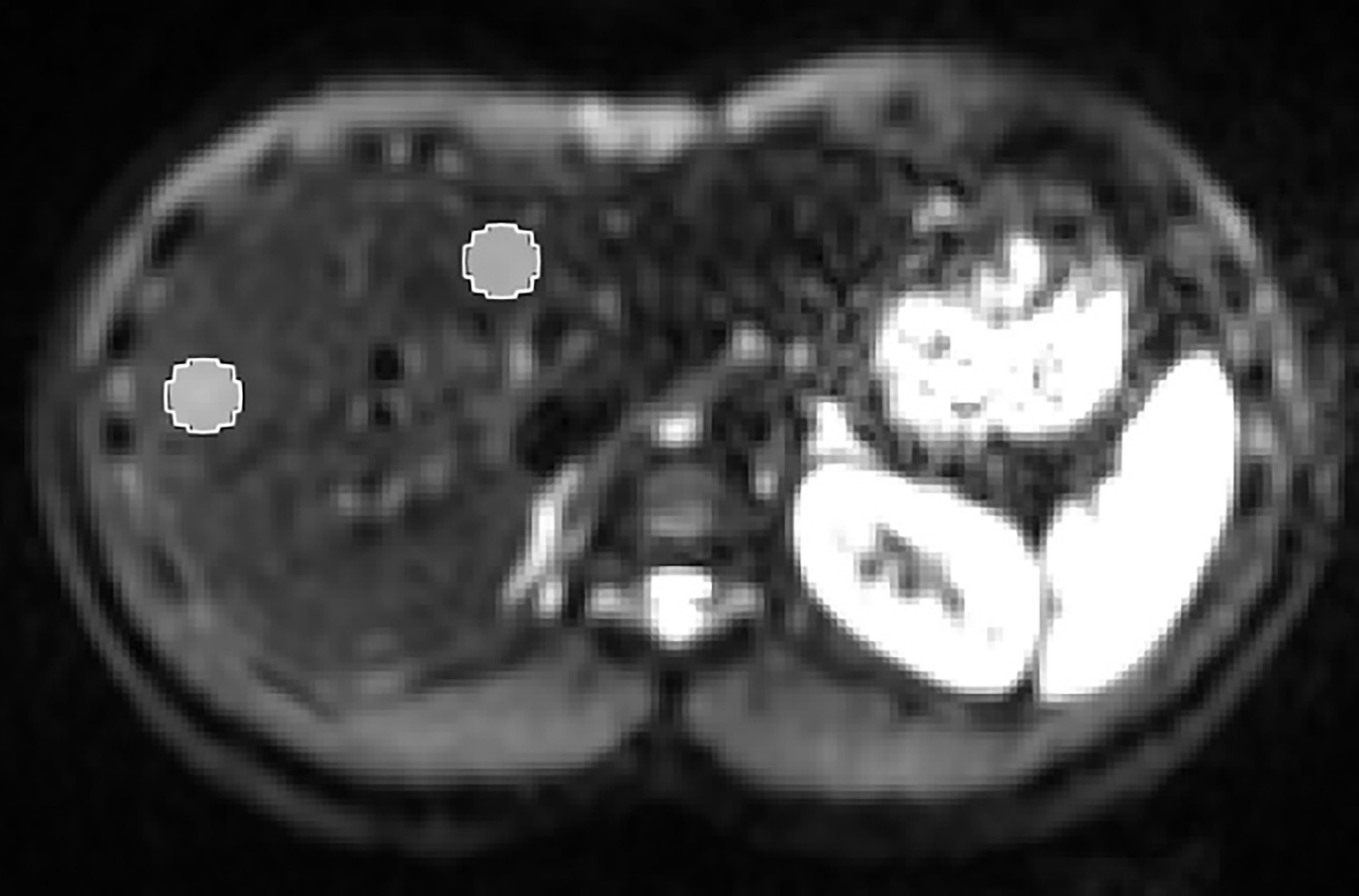
Representative segmentations for signal calculation in the left and right liver lobe.

The evaluation was performed on the trace-weighted images. They were calculated by averaging the five repetitions per diffusion direction arithmetically and then averaging the resulting three images geometrically. To evaluate the signal loss, two circular regions with a diameter of 10 voxels (3.5 cm) were segmented in all slices showing both liver lobes; one region was specified in the left liver lobe and one was in the right liver lobe. We took care not to include vessels or signal from fat. Representative segmentations are shown in Fig 2.

For each diffusion encoding and segmentation, the mean signal of the included voxels was calculated and normalized on the mean signal of the right liver lobe in the monopolar b = 50 s/mm^2^ image (of the same slice). Then, the mean signal of the left and right lobe was calculated for each diffusion encoding as the average over all slices.

**Fig 3:**
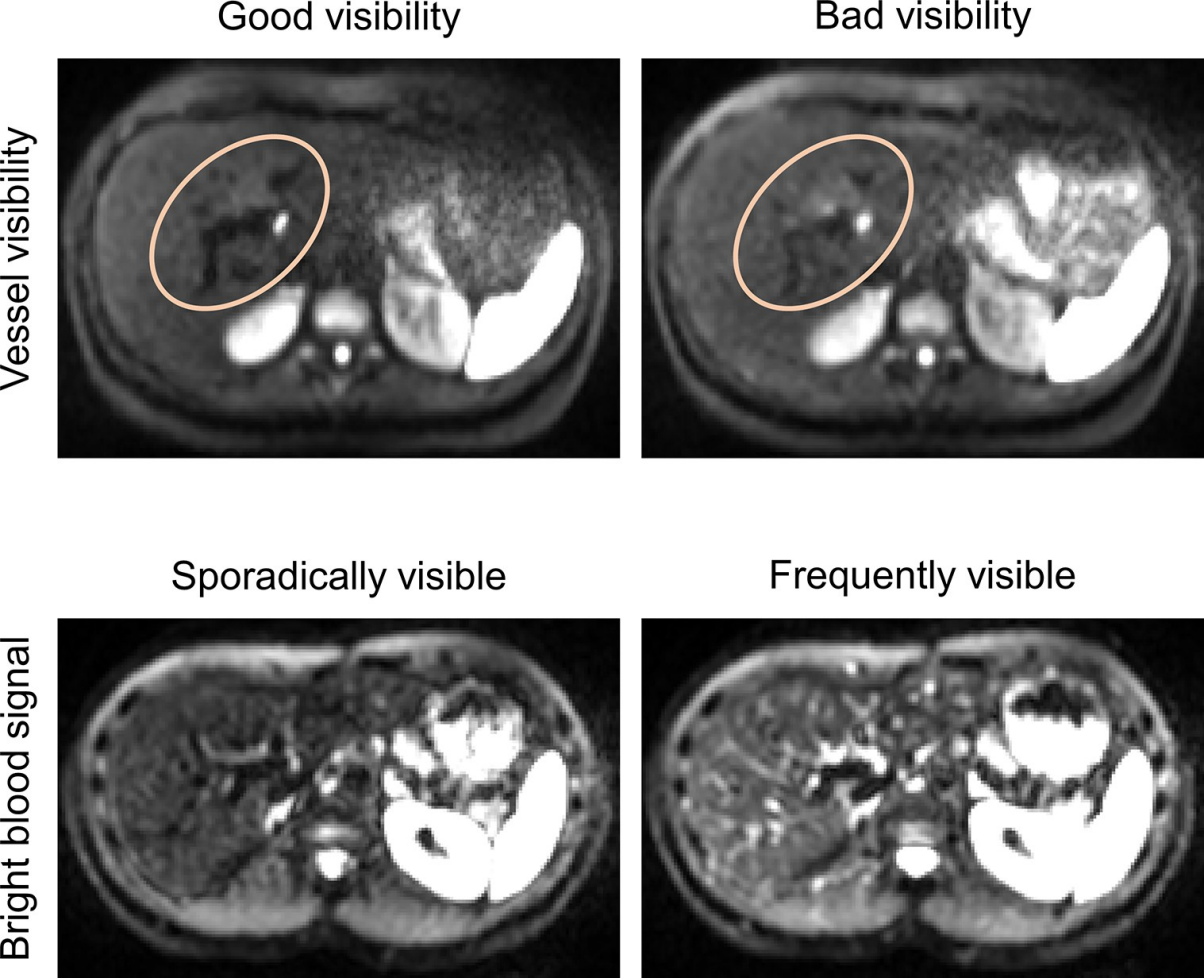
Examples for the evaluated properties “vessel visibility” and “bright blood signal”.

The blood appearance was assessed by two properties: First, the visibility of vessels compared to the liver parenchyma was assessed. This property describes the contrast between vessels and tissue and aims at describing the presence or loss of anatomical landmarks due to an isointensity of vessels and liver parenchyma. Second, the frequency of bright blood signals, appearing as bright spots, was rated. This property refers to the occurrence of bright spots in small vessels or at the edge of large vessels, which mimic lesions and might potentially lead to false positive lesions. Explanatory example images are shown in Fig 3. For each property, a 5-point Likert scale was used. The definition of the scores is shown in Table 3.

**Table 3:** Likert scales for the black blood state evaluation.

	Bright blood signal	Vessel visibility
1	Bright blood visible in all slices	No vessels identifiable
2	Bright blood visible in most slices	Vessels hardly identifiable
3	Bright blood visible in some slices	Vessels sometimes not well visible
4	Bright blood sporadically visible	Vessels identifiable in most slices
5	Bright blood not significantly visible	Vessels very well identifiable in all slices

The reading was performed in a blinded fashion by a physicist with four years of experience in abdominal MRI.

For each of the seven volunteers, the *b* = 50 s/mm^2^ images were considered first. For each diffusion encoding, the two properties were rated considering the whole set of acquired slices. Second, the *b* = 800 s/mm^2^ images were rated analogously.

Afterwards, the scores were averaged across volunteers.

For the ADC assessment, ADC maps were created from the b = 50 s/mm^2^ and the b = 800 s/mm^2^ images. The ADC was calculated as the mean ADC in the segmentations and finally averaged over all slices.

We performed statistical tests to find differences in the signal ratios between the left and right lobe, in the Likert scores for the blood appearance evaluation, and in the ADC values using the Kruskal-Wallis test. As posthoc test, the Dunn test with Bonferroni correction was used. The significance level was set to 0.05.

## Results

**Fig 4:**
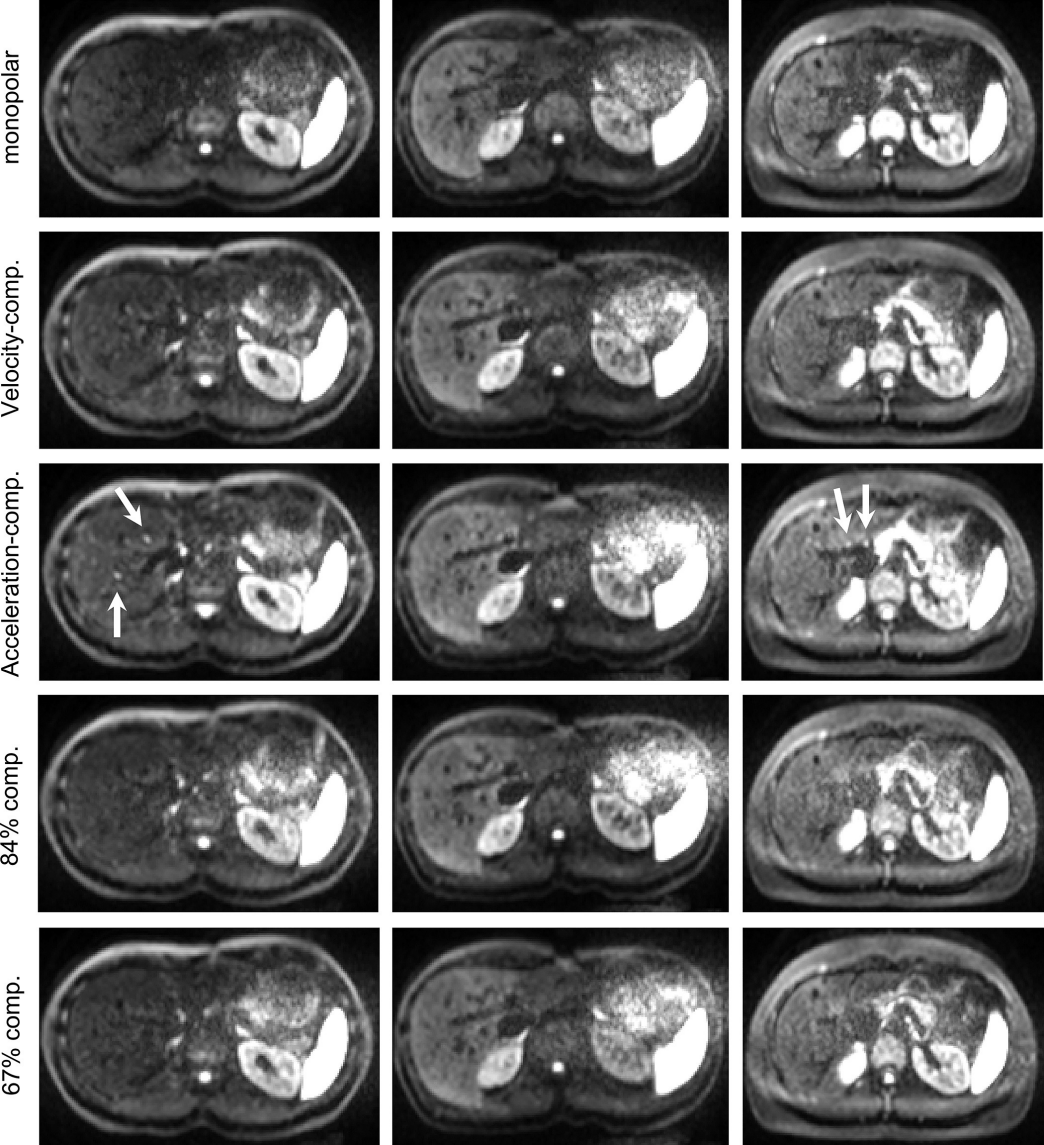
Trace-weighted images at b = 800 s/mm^2^. Each column shows the same slice. Bright blood signals are depicted by white arrows.

**Fig 5:**
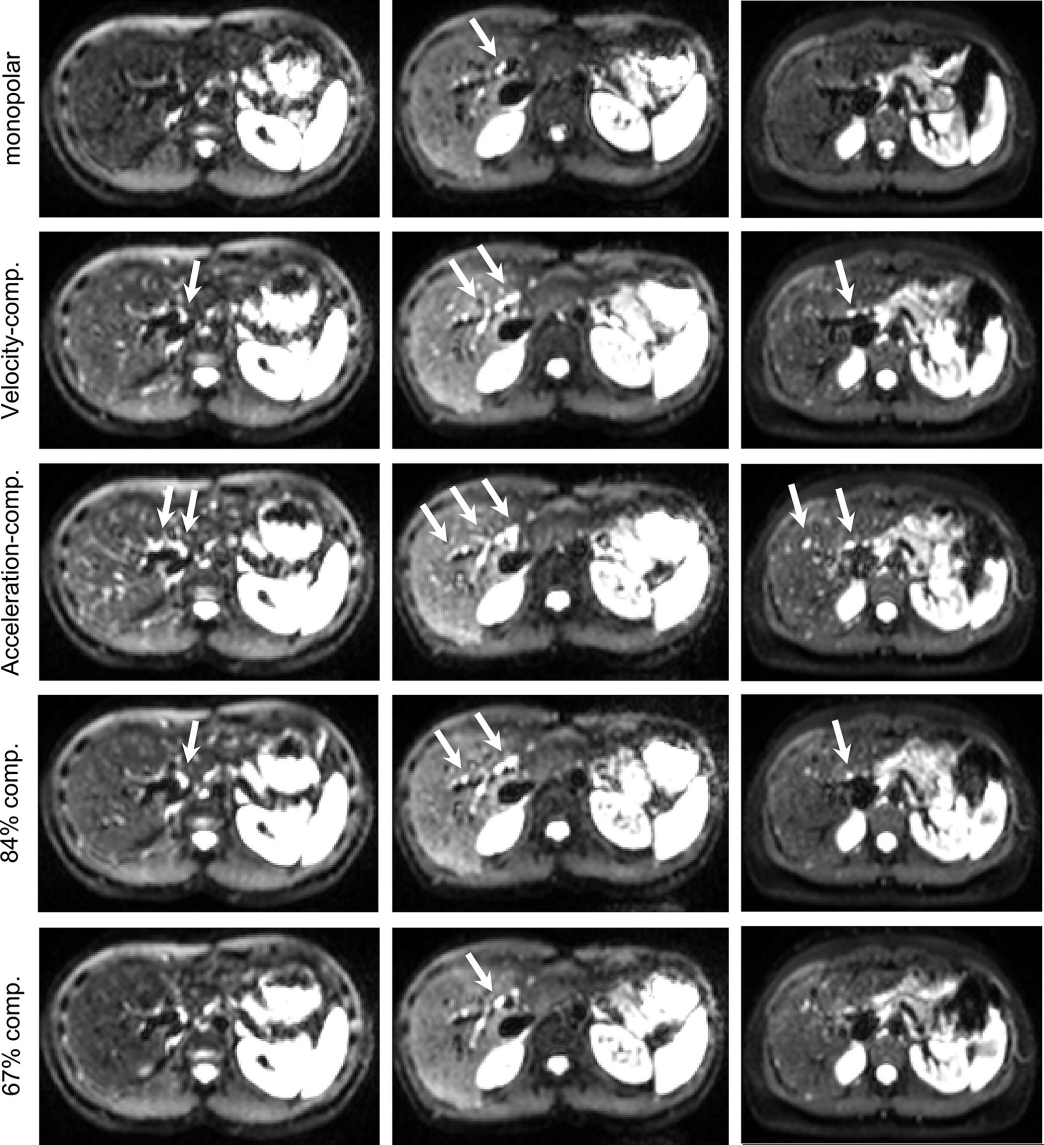
Trace-weighted images at b = 50 s/mm^2^. Each column shows the same slice, same slices as in Fig 2. Bright blood signals are depicted by white arrows.

Fig 4 shows representative trace-weighted images at b = 800 s/mm^2^ for the five diffusion encodings depicted in Fig 1. The images indicate an increased signal for velocity- and acceleration-compensated encodings, especially in the left liver lobe. For the 84%- and 67%-compensated encodings, the signal decreases slightly, again with stronger decrease in the left liver lobe. Signal from blood vessels is highest in the acceleration-compensated images. Fig 5 displays the respective images at b = 50 s/mm^2^. The bright blood spots are more prevalent than at b = 800 s/mm^2^, especially for acceleration-compensation, but also to lesser extent for velocity-compensated, 67%-, and 84%-compensated encodings. The signal decrease in the left liver lobe is much reduced compared to b = 800 s/mm^2^.

**Fig 6:**
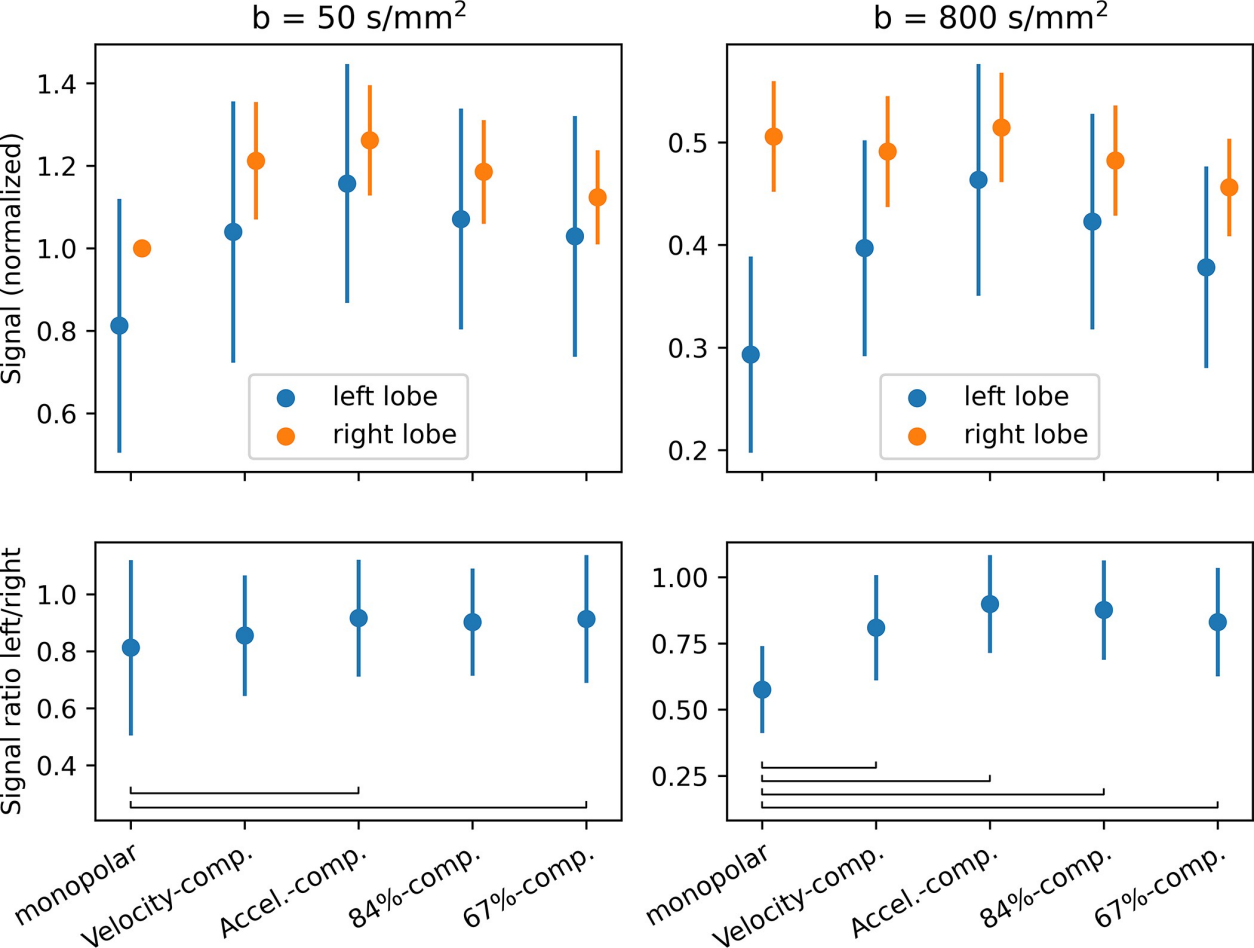
Signals (top) Mean signals in the left and right liver lobe for b = 50 and b = 800 s/mm^2^, normalized to the monopolar signal at b = 50 s/mm^2^ in the right lobe and averaged over all slices. The error bars represent the standard deviation of the averaged signals among slices and volunteers. (bottom) Ratio of the signal in the left and right liver lobe. Brackets indicate significant differences.

The mean signal for both b-values in the left and right liver lobes is shown in Fig 6 and in Table 4 (normalized to the slice-averaged monopolar b = 50 s/mm^2^ signal). Overall, the signal is higher in the right than in the left liver lobe for all diffusion encodings and b-values. The signal is highest for the acceleration-compensated diffusion encoding. In the left liver lobe, the signal is lowest for the monopolar encoding. In the right liver lobe, the signal changes are generally smaller, especially at b = 800 s/mm^2^. The relative signal difference between the left and right lobe is largest for the monopolar encodings with 19% and 42% for b = 50 s/mm^2^ and 800 s/mm^2^, respectively, and smallest for the acceleration-compensated encodings with 8% and 10%, respectively. For 84%- and 67%-compensation, the difference is slightly larger (10%/13% and 9%/16%), but still smaller than for the velocity-compensated encodings (14%/19%).

**Table 4:** Mean and standard deviation of the signals in the left and right liver lobe for b = 50 and b = 800 s/mm^2^, normalized to the monopolar signal at b = 50 s/mm^2^ in the right lobe and averaged over all slices.

	*b* = 50 s/mm^2^		*b* = 800 s/mm^2^	
	Left lobe	Right lobe	Left lobe	Right lobe
monopolar	0.81±0.31	1	0.29±0.10	0.51±0.05
Velocity-comp.	1.04±0.32	1.21±0.14	0.40±0.11	0.49±0.05
Accel-comp.	1.16±0.29	1.26±0.13	0.46±0.11	0.51±0.05
84%-comp.	1.07±0.27	1.19±0.13	0.42±0.10	0.48±0.05
67%-comp.	1.03±0.29	1.12±0.11	0.38±0.10	0.46±0.05

**Fig 7:**
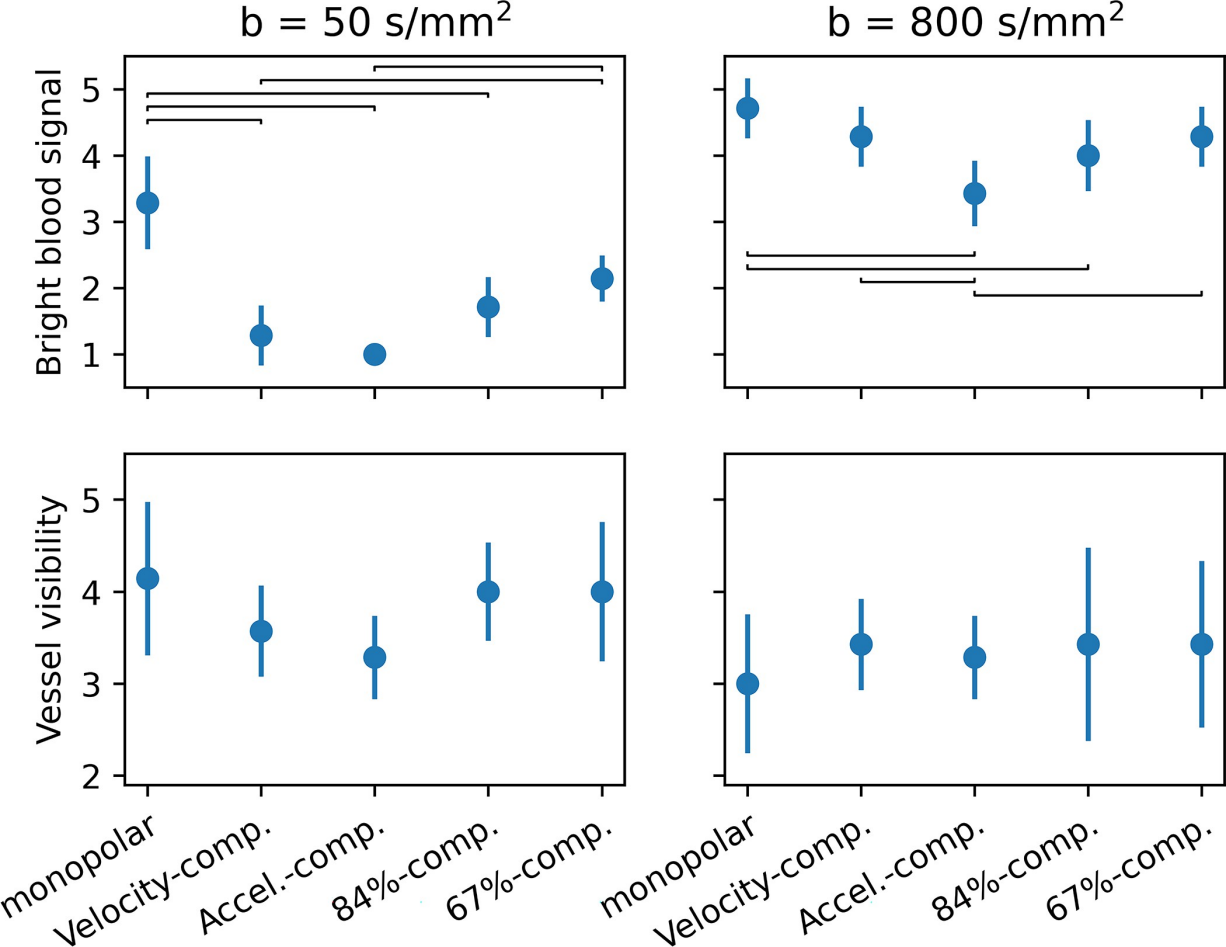
Averaged Likert scores for the blood appearance evaluation. Note the definitions in Table 3 (i.e., a lower score for the bright blood signal means a brighter blood signal). Brackets indicate significant differences.

Fig 7 and Table 5 show that bright blood signals are most frequent for the *M*_2_-compensated encoding and least frequent for the monopolar encoding. This is valid for both b-values. With the partially compensated encodings, the scores increase again but do not reach the level of the monopolar encoding. The dependence of the vessel visibility on the diffusion encoding is less pronounced, differing between *b* = 50 and *b* = 800 s/mm^2^. For both b-values, the partially compensated encodings perform well in this regard.

**Table 5:** Averaged Likert scores for the blood appearance evaluation.

	*b* = 50 s/mm^2^		*b* = 800 s/mm^2^	
	Bright blood signal	Vessel visibility	Bright blood signal	Vessel visibility
monopolar	3.29±0.70	4.14±0.83	4.71±0.45	3.00±0.76
Velocity-comp.	1.29±0.45	3.57±0.49	4.29±0.45	3.43±0.49
Accel-comp.	1±0	3.29±0.45	3.43±0.49	3.29±0.45
84%-comp.	1.71±0.45	4.00±0.53	4.00±0.53	3.43±1.05
67%-comp.	2.14±0.35	4.00±0.75	4.29±0.45	3.43±0.90

Note the definitions in Table 3 (i.e., a lower score for the bright blood signal means a brighter blood signal).

**Fig 8:**
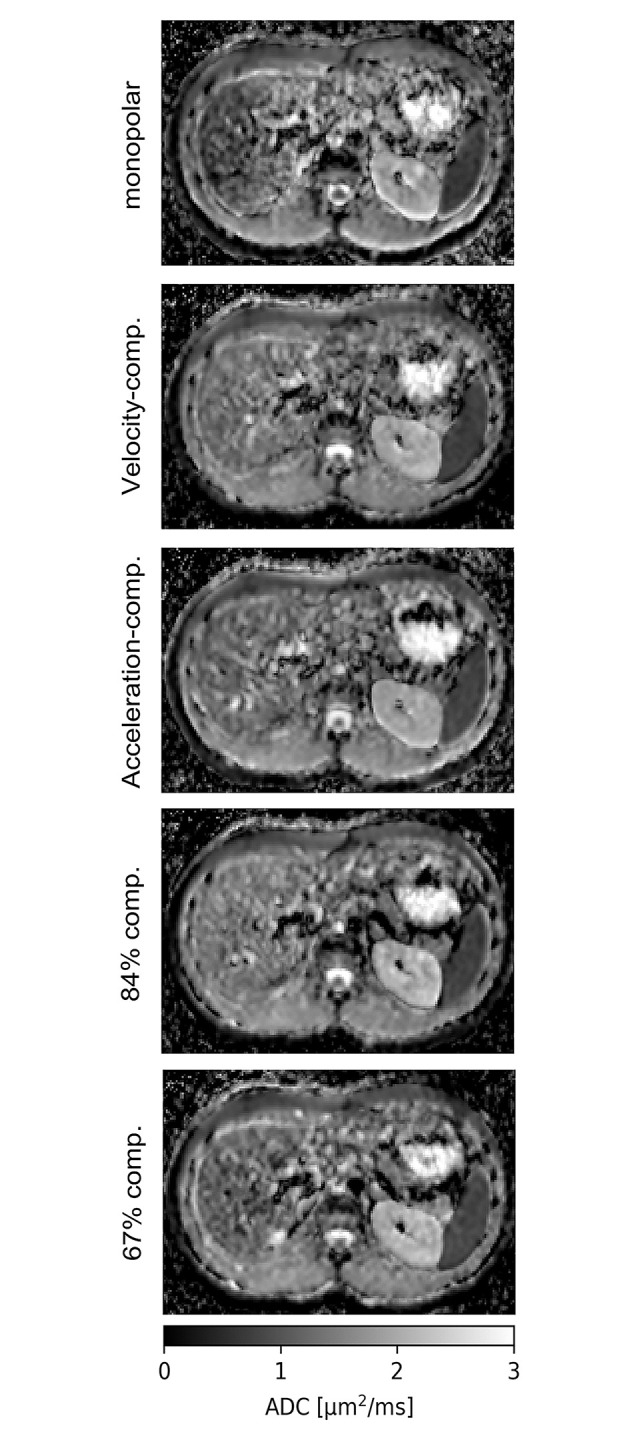
ADC maps of one volunteer.

**Fig 9:**
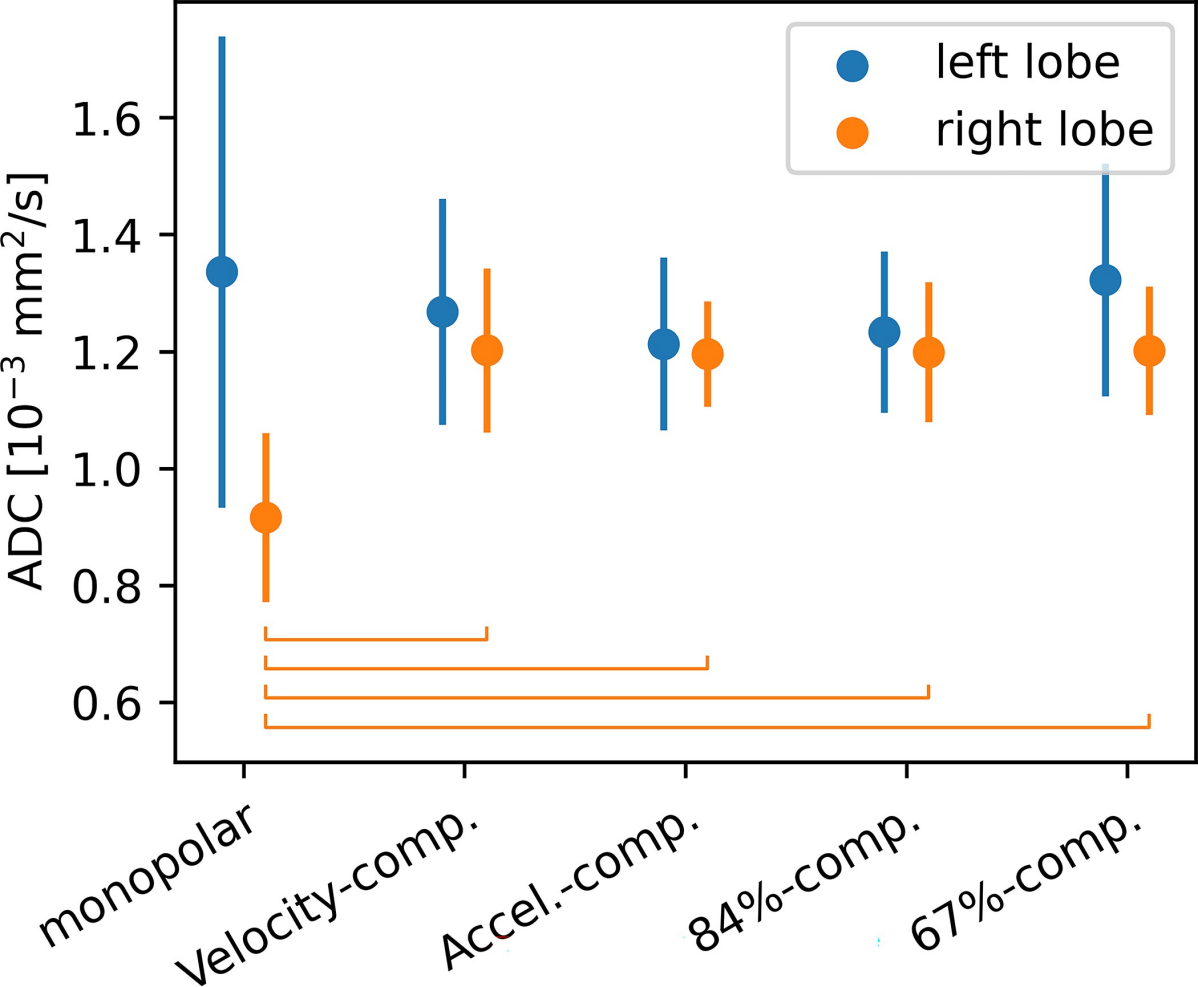
ADC values in the left and right liver lobe, averaged over all slices. Error bars represent the standard deviation among all slices and volunteers. Brackets indicate significant differences.

ADC maps for the different encoding schemes are shown in Fig 8. The behavior of the ADC values (see Fig 9, Table 6) differs strongly between left and right liver lobe. In the left liver lobe, the ADC is lowest for the acceleration-compensated encodings and highest for the monopolar and the 67%-compensated encodings. In the right lobe, the ADC is nearly constant for all diffusion encodings except for the monopolar one, which yields a much lower ADC. In general, the ADC is higher in the left lobe, and the difference between the left and right lobe is the smallest for the acceleration-compensated encoding.

**Table 6:** ADC values in μm^2^/ms in the left and right liver lobe, averaged over all slices.

	Left lobe	Right lobe
monopolar	1.33±0.40	0.92±0.14
Velocity-comp.	1.27±0.19	1.20±0.14
Accel-comp.	1.21±0.15	1.20±0.09
84%-comp.	1.23±0.18	1.20±0.12
67%-comp.	1.32±0.20	1.20±0.11

## Discussion

In this work, we evaluated different diffusion encoding schemes with respect to their abilities to reduce signal loss in the left liver lobe, their influence on ADC values, and with respect to the appearance of blood signal.

We showed that acceleration-compensated encodings also worked reliably and did not induce unexpected image impressions for a b-value of 800 s/mm^2^, while the largest previously tested b-value in the liver for this scheme was 500 s/mm^2^ to the best of our knowledge [15, 16]. This indicates that a potential clinical use with b = 800 s/mm^2^, which has been used in many previous liver MRI studies [32–35], is possible. Furthermore, the 84%- and 67%-compensated encodings, created in this study with a novel technique, worked reliably.

The main finding is a contrary effect between the signal loss in the left liver lobe and the black-blood state. Blood looked darkest for the monopolar encodings (Bright blood scores: 3.3 and 4.7), whereas the signal loss was highest (Signal loss: 19% and 42%). For the acceleration-compensated encodings, the signal loss was lowest (Signal loss: 8% and 10%), but the blood appeared hyperintense (Bright blood scores: 1.0 and 3.4). Therefore, the results indicate that a single optimal level of motion encoding might not exist. An optimal level could exist if the distribution of blood velocities were much broader than the distribution of the velocities in the liver. Then, a small velocity-weighting would lead to substantial attenuation of the blood signal, while the rather coherent liver motion would not induce large signal loss. However, this did not seem to be the case.

The finding that the signals for b = 800 s/mm^2^ in the right liver lobe did not substantially change with different encodings implies no strong influence by first- or second-order motion in the right liver lobe, which can be induced by cardiac or breathing motion. We did not expect the latter to occur, however, because the measurements were performed during breath-hold in expiration. The signal increase in the left liver lobe from the monopolar encodings to *M*_1_-compensated encodings (by about 30%) and further to *M*_2_-compensated encodings (by about 50%) indicates that a substantial proportion of the signal loss is due to second-order motion, which was also reflected in the signal ratios. As the minimal relative signal difference between the left and right lobe was not more than 10%, we conclude that most of the liver motion due to cardiac pulsation could be described by motion up to the second order. In other words, compensation of higher orders of motion would maximally restore less than 10% of the signal. Third-order motion-compensation does therefore not appear necessary.

However, as expected, the acceleration-compensated encodings also increased the bright blood signal and decreased the overall vessel visibility. This is in line with the report by Zhang et al. [15], who used b = 500 s/mm^2^. In terms of blood suppression, fully *M*_2_-compensated encodings are therefore not recommended.

The 84%- and 67%-compensated encodings aimed to overcome the black-blood limitation of the acceleration-compensated encoding and suppressed the blood signal quite well, which was a reasonable result. In many DWI liver studies, 50 s/mm^2^ (instead of 0 s/mm^2^) is used as small b-value to suppress bright blood signal [36–38]. With usual monopolar waveforms at 70 ms echo time, this approximately refers to an *M*_1_-weighting of 1.5 s/mm. Our 84% and 67% compensated waveforms at b = 800 s/mm^2^ induce a velocity-weighting of about 0.9 s/mm and 1.9 s/mm, respectively. Thus, our observations of increasing blood signal suppression coincide quite well with the expected behavior. Zhang et al. [15] used flow-weightings of 0.6 s/mm and 1.3 s/mm and also reported that both show substantial blood signal suppression (they stated values of 0.1 s/mm and 0.2 s/mm, but used a value of *γ* = 42.58 MHz/T, which is actually *γ*/(2*π*).) They did not observe a difference between both flow-weightings. This might be explained by an effect similar to the triexponential intravoxel incoherent motion (IVIM) behavior [39–42], i.e., faster flowing blood is of the highest relevance and is suppressed at even smaller weightings. However, our findings do not reveal whether the blood signal is reduced stronger because of the *M*_1_-weighting increase or because of the *M*_2_-weighting increase.

The ADC values in the left liver lobe behaved as expected; the signal at b = 800 s/mm^2^ was higher for the acceleration-compensated encodings, therefore the ADC was lower (1.21 μm^2^/ms) than for the monopolar encodings (1.33 μm^2^/ms), where a slight overestimation occured. In the right liver lobe, the behavior was more interesting. We expected the ADC to be relatively independent on the diffusion encoding because signal loss (and its compensation) is more common in the left liver lobe. However, the ADC was much lower for the monopolar encodings (0.92 μm^2^/ms) compared to the others (1.20 μm^2^/ms). With no remarkable difference in the signals at b = 800 s/mm^2^, the difference must be due to a lower signal of the monopolar encoding at b = 50 s/mm^2^. As the signal dropout due to pulsation predominantly occurs at higher b-values, this signal loss must be induced by a different mechanism. This phenomenon is most likely due to IVIM effects [39, 40]: In the ballistic limit, the blood in the single vessels does not change its direction during measurement. While this induces signal loss in the monopolar measurement, the signal loss is (partly) compensated in the other measurements, where a certain degree of velocity-compensation is employed. This assumption is confirmed by Moulin et al. [21], who showed that the signal at low b-values strongly depends on the strength of the velocity weighting in IVIM measurements. In the ballistic regime, velocity compensation works to retain the signal that is otherwise dephased from incoherent flow, leading to a higher ADC. Using *D*_blood_≈1.6 μm^2^/ms [43] and *D*_liver_≈0.95 μm^2^/ms [42], our measured value of 0.92 μm^2^/ms appears reasonable for the monopolar case, as the blood is attenuated in this case. For the other encodings, the blood signal is not fully attenuated and one can estimate the measured ADC as *f*∙*D*_blood_+(1−*f*)∙*D*_liver_. With *f*≈0.3 at TE = 70 ms [42], the thus estimated ADC is 1.17 μm^2^/ms and corresponds well to the measured value of 1.20 μm^2^/ms for the flow-compensated case.

Geng et al. [22] showed that gradient waveforms with small *M*_1_ values combined with segmented echo planar readouts are well-suited to obtain low-distortion images without a large influence from motion artifacts in liver DWI. The application of small *M*_2_ values may be an interesting further step in this regard. Additionally, Kwee et al. reported that the systolic signal is lower than the diastolic signal, which suggests that the strength of the signal dropout depends on the cardiac cycle [8]. This supports using an ECG trigger to only measure during the optimal point of the cardiac cycle [44, 45]. However, this prolongs acquisition times, and the strong gradients may render the triggering unreliable [46]. The authors also showed that the signal loss in the left lobe is more pronounced for superior-inferior diffusion-sensitizing gradients, whereas this holds true for the left-right direction in the right lobe. Based on this finding, Van et al. tried to use velocity-compensated encodings only on some gradient axes [47]. Similarly, Ozaki et al. used velocity-compensated gradient waveforms but in a set of tetrahedral directions. They showed that the motion artifact could be reduced best with this setup [9]. McTavish et al. [48] reported that care needs to be taken when applying gradient nonlinearity correction methods, as the ADC bias may also increase. Moreover, it appears reasonable that the strength of the signal dropout is dependent on the breathing cycle and stronger during exhalation than inhalation, because the distance between the liver and the heart changes. However, Riexinger et al. found no significant difference in the data quality between inhalation and exhalation [11]. The study here on the liver with the emphasis on its laterality might also have implications for the kidneys where diffusion-weighted processing pipelines [49] separately process the left and right kidneys due to their differences in motion, and signal dropouts.

In some studies, an aim was to minimize the echo time for each diffusion encoding separately [16, 22, 23]. For example, Aliotta et al. proposed the CODE optimization algorithm to create asymmetric motion-compensated waveforms and demonstrated a significant reduction in the echo time [23]. In our study, we kept TE fixed on purpose, however, to determine how different encodings affect normalized signals. The commonly named reason for the minimization of TE is to improve the signal-to-noise ratio. Still, as Laun et al. [19] pointed out, TE should be chosen instead to maximize the T2w-contrast between tissue and possible lesions. With this contrast maximizing condition, they reported an optimal TE of about 67 ms at 1.5 T. This suggests that it might not be necessary to minimize the TE as much as possible.

In addition to avoiding unwanted signal dropout during the measurement, it is also possible to use post-processing techniques. Several algorithms have been proposed, which are mostly based on outlier rejections or a greater weighting for higher signals during the averaging of repetitions [50–52], sometimes combined with deep learning [53, 54]. In a quantitative comparison, the choice of algorithm parameters has been shown to be non-trivial, indeed affecting the outcome [20].

We acknowledge several limitations of this study: First, the *M*_1_ and *M*_2_ values for the different encodings are dependent on the b-value and not kept constant, which may be considered a drawback of the proposed waveform calculation approach. However, this approach is advantageous in terms of time-efficiency, as it eliminates the need to create a new waveform for each b-value. Alternatively, the proposed encodings may only be used for the higher b-value, while for the low b-value, monopolar encodings can be used, as proposed by Rauh et al. [10], which would mitigate this limitation. Second, the study was limited by the small number of volunteers [55] and the use of a single scanner, which might have affected the generalizability of the results. Third, it might have been better to use a higher b-value (like 100 s/mm^2^) instead of 50 s/mm^2^ to achieve a better blood suppression. Finally, the uniform echo times for all encodings were only achievable because of to the high-performing gradients of the scanner used. This may pose a challenge in clinical settings where the available gradients strengths may not be as high. Conversely, in practice, the monopolar diffusion encoding would presumably be acquired with the shortest possible echo time, which may be shorter than the used echo time of 70 ms.

In conclusion, this study showed that easy-to-implement gradient waveforms with adjustable compensation levels of *M*_1_ and *M*_2_ are well-suited even for b-values of b = 800 s/mm^2^. We showed that the blood signal does not only depend on the *M*_1_ weighting but also on the *M*_2_ weighting. The results indicate that it is difficult to find a sweet spot that minimizes the blood signal and maximizes the signal in the left liver lobe at the same time.

## Supporting information

S1 File Full calculations for *M*_1_ and *M*_2_ and visualization of parameters. (PDF)
