# Supplementary material for: Impact of velocity- and acceleration-compensated encodings on signal dropout and black-blood state in diffusion-weighted magnetic resonance liver imaging at clinical TEs
Source: PLoS One. 2023 Oct 5;18(10):e0291273. doi: 10.1371/journal.pone.0291273 (PMC10553293; doi:10.1371/journal.pone.0291273)
Supplement: S1 File — (PDF) [file pone.0291273.s001.pdf]

Supporting Information

<u>Velocity-compensated gradient pulses</u>

Consider a given gradient pulse $G_{\text{pulse}}(t)$ with the duration t_1.

A gradient profile $G(t)$ is created from this pulse as follows:

$$G(t) = \begin{cases} G_{\text{pulse}}(t) & \text{for } 0 < t < t_1 \\ -G_{\text{pulse}}(t-t_1) & \text{for } t_1 < t < 2\,t_1 \\ 0 & \text{for } 2t_1 < t < 2t_1 + t_2 \\ -G_{\text{pulse}}(t-(2t_1+t_2)) & \text{for } 2t_1 + t_2 < t < 3t_1 + t_2 \\ G_{\text{pulse}}(t-(3t_1+t_2)) & \text{for } 3t_1 + t_2 < t < 4t_1 + t_2 \end{cases}$$

The velocity-weighting M_1 is calculated via

$$M_1 = \gamma \int_0^{TE} G(t) \cdot t\,dt$$

$$= \gamma \left[\int_0^{t_1} G_{\text{pulse}}(t) \cdot t\,dt - \int_{t_1}^{2t_1} G_{\text{pulse}}(t-t_1) \cdot\ t\,dt\ - \int_{2t_1+t_2}^{3t_1+t_2} G_{\text{pulse}}\big(t-(2t_1+t_2)\big) \cdot t\,dt + \int_{3t_1+t_2}^{4t_1+t_2} G_{\text{pulse}}\big(t-(3t_1+t_2)\big) \cdot\ t\,dt \right]$$

Substituting $t' = t - t_1, t'' = t - (2t_1 + t_2)$, and $t''' = t - (3t_1 + t_2)$ yields

$$M_1 = \gamma \left[\int_0^{t_1} G_{\text{pulse}}(t) \cdot t\,dt - \int_0^{t_1} G_{\text{pulse}}(t') \cdot (t' + t_1) dt' - \int_0^{t_1} G_{\text{pulse}}(t'') \cdot \big(t'' + (2t_1 + t_2)\big) dt'' + \int_0^{t_1} G_{\text{pulse}}(t''') \cdot \big(t''' + (3t_1 + t_2)\big) dt''' \right]$$

$$= \ \gamma \left[\int_0^{t_1} G_{\text{pulse}}(t) \cdot t\,dt - \int_0^{t_1} G_{\text{pulse}}(t') \cdot t' dt' - \int_0^{t_1} G_{\text{pulse}}(t') \cdot t_1\,dt' - \int_0^{t_1} G_{\text{pulse}}(t'') \cdot t'' dt'' - \int_0^{t_1} G_{\text{pulse}}(t'') \cdot (2t_1 + t_2)\,dt'' + \int_0^{t_1} G_{\text{pulse}}(t''') \cdot t'''\,dt''' + \int_0^{t_1} G_{\text{pulse}}(t''') \cdot (3t_1 + t_2)\,dt''' \right]$$

The four integrals over $G_{\text{pulse}}(t) \cdot t$ cancel, because they are integrated over the same time range. The other integrals (over $G_{\text{pulse}}(t)$) can be combined as they are also integrated over the same time range:

$$M_1 = \gamma[-t_1 - (2t_1 + t_2) + (3t_1 + t_2)] \cdot \int_0^{t_1} G_{\text{pulse}}(t)\,dt = 0$$

Acceleration-compensated gradient pulses

Consider a gradient profile of the following shape:

$$G(t) = G \cdot \begin{cases} 1 & \text{for } 0 < t < a \\ -1 & \text{for } a < t < a+b \\ 0 & \text{for } a+b < t < a+b+c \\ 1 & \text{for } a+b+c < t < a+2b+c \\ -1 & \text{for } a+2b+c < t < 2a+2b+c \end{cases}$$

where $a < b$, $a, b, c > 0$.

$$M_2 = \gamma \int_0^{TE} G(t) \cdot t^2 \, dt$$

$$= \gamma G \left[\int_0^a t^2 \, dt - \int_a^{a+b} t^2 \, dt + \int_{a+b+c}^{a+2b+c} t^2 \, dt - \int_{a+2b+c}^{2a+2b+c} t^2 \, dt \right]$$

$$= \frac{\gamma G}{3} [a^3 - ((a+b)^3 - a^3) + ((a+2b+c)^3 - (a+b+c)^3) - ((2a+2b+c)^3 - (a+2b+c)^3)]$$

$$= \gamma G[-2a^3 + 2b^3 - 6a^2 b - 3a^2 c + 3b^2 c - 2ab^2 - ac^2 + bc^2 - 2abc]$$

The equation $M_2 = 0$ can be solved analytically by rearranging the above formula into a quadratic formula for c.

$$M_2 = \gamma G[(b-a)c^2 + (3b^2 - 2ab - 3a^2)c \; + \; 2b^3 - 2a^3 - 6a^2 b - 2ab^2]$$

Thus, for $M_2 = 0$,

$$c = \frac{-(3b^2 - 2ab - 3a^2) \pm \sqrt{D}}{2(b-a)}$$

with

$$D = (3b^2 - 2ab - 3a^2)^2 - 4(b-a)(2b^3 - 2a^3 - 6a^2 b - 2ab^2)$$

$$= (b^2 + 2ab - a^2)^2$$

must hold true.

The formula for c can be simplified if $(b^2 + 2ab - a^2)$ is positive, which implies

$$b < \left(1 + \sqrt{2}\right) a.$$

Then,

$$c = \frac{-(3b^2 - 2ab - 3a^2) \pm (b^2 + 2ab - a^2)}{2(b-a)}$$

The „-“ solution, $-2(a+b)$, yields only negative values. The “+” solution reads:

$$c = \frac{-b^2 + 2ab + a^2}{b-a}$$

c is negative for $b < a$ and for $b > (1 + \sqrt{2})a$. Otherwise, it is positive. Thus, for a given gradient duration a, the gradient duration b must be within $(a, (1 + \sqrt{2})a)$. The pause duration c ensuring acceleration-compensation can then be calculated with the above formula.

This $G(t)$ is also M_1-compensated:

$$M_1 = \gamma G[-a^2 - 2ab - ac + b^2 + bc] = \gamma[c(b - a) + b^2 - 2ab - a^2] = 0.$$

In the last step, the equation for c was inserted.

Replacing a by $a + t_S$ and b by $b - t_S$ and rearranging for an expression of t_S yields:

$$M_2(t_S) = \gamma G[-2\ (2b + c)(2a + 2b + c) \cdot t_S + 2(2a + 2b + c) \cdot {t_S}^2]$$

$$M_1(t_S) = \gamma G[-2(2b + c) \cdot t_S + 2 \cdot {t_S}^2]$$

The first order term vanished because c was assumed to equal $(-b^2 + 2ab + a^2) \cdot (b - a)^{-1}$.

To achieve the same b-value, there is also a slight variation in the gradient amplitude G necessary:

The formula for the b-value of a gradient pulse of the above shape can be calculated as:

$$b_{\text{diff}} = \frac{\gamma^2 G^2}{3} \cdot (2a^3 + 6a^2 b - 6ab^2 + 2b^3 + 3a^2 c + 3b^2 c - 6abc)$$

Replacing a by $a + t_S$ and b by $b - t_S$ and rearranging in terms of t_S yields

$$b_{\text{diff}}(t_S) = \frac{\gamma^2 G^2}{3}\big[(2a^3\ +\ 3a^2(2b + c) - 6ab(b + c) + b^2(2b + 3c)) + 12\big(a(2b + c) - b(b + c)\big) \cdot t_S\ - 12(a - 2b - c) \cdot {t_S}^2 - 12 \cdot {t_S}^3\ \big]$$

Solving for G yields

$$G(t_S) = \frac{\sqrt{b_{\text{diff}}}}{\gamma} \cdot \sqrt{\frac{3}{[\ldots]}}$$

and inserting in the formula for M_2:

$$M_2(t_S) = \sqrt{3 b_{\text{diff}}} \frac{-2\ (2b + c)(2a + 2b + c) \cdot t_S + 2(2a + 2b + c) \cdot {t_S}^2}{\sqrt{[\ldots]}}$$

Taylor expanding in t_S yields:

$$M_2(t_S) = \sqrt{3b_{\mathrm{diff}}} \frac{-2\,(2b+c)(2a+2b+c)\cdot t_S}{\sqrt{2a^3 \;+\; 3a^2(2b+c) - 6ab(b+c) + b^2(2b+3c)}} + O({t_S}^2)$$

For M_1:

$$M_1(t_S) = \sqrt{3b_{\mathrm{diff}}} \frac{-2\,(2b+c)\cdot t_S}{\sqrt{2a^3 \;+\; 3a^2(2b+c) - 6ab(b+c) + b^2(2b+3c)}} + O({t_S}^2)$$

These calculations are valid for rectangular gradient shapes. For realistic trapezoidal shapes, the behavior of both M_1/M_1^{max} and M_2/M_2^{max} is nearly equal, as it can be seen in Fig. A1. $M_{1/2}^{\mathrm{max}}$ refers to $M_{1/2}$ of the respective monopolar gradient pulse.

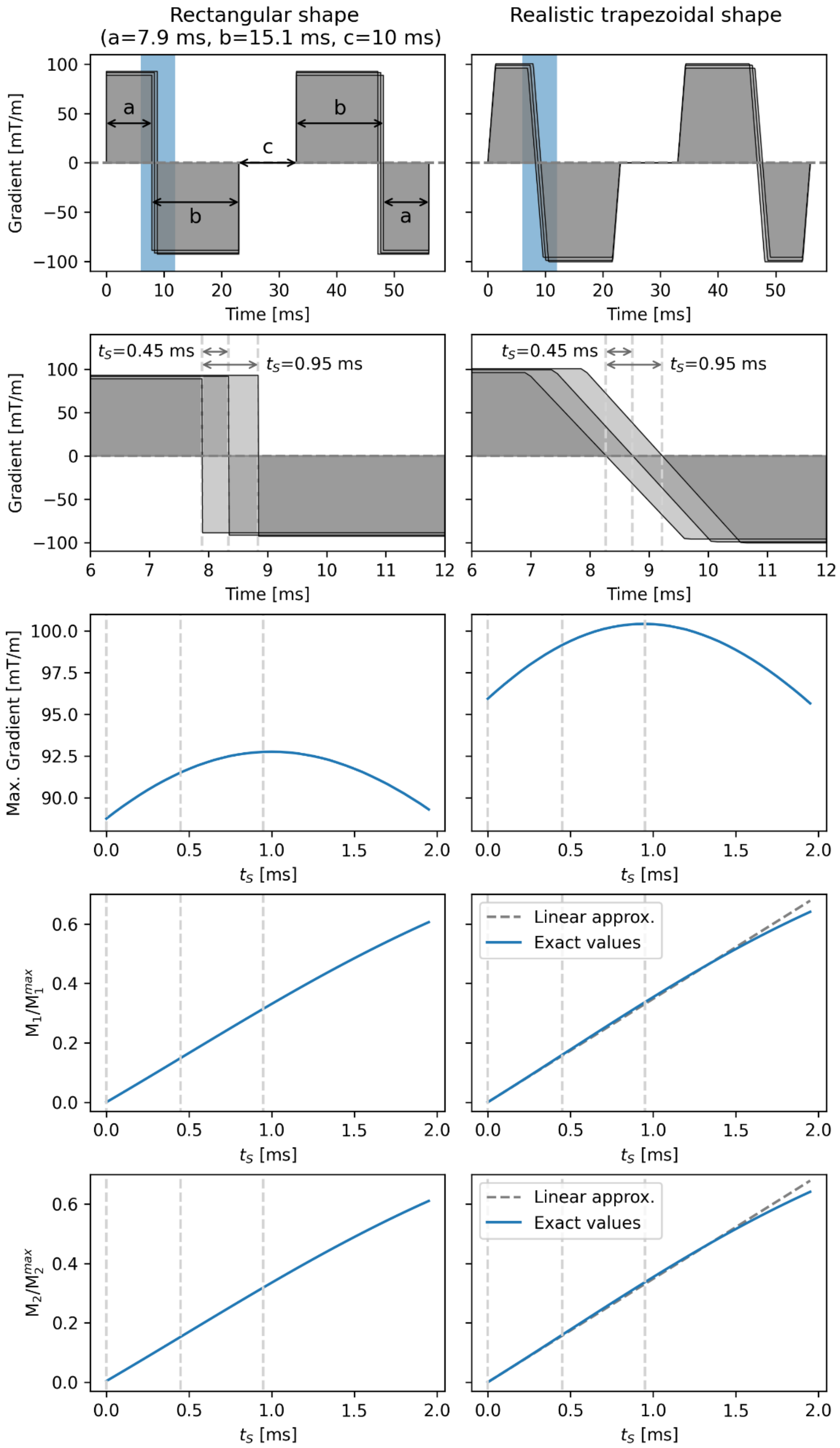

S1 Fig: Comparison of the idealized rectangular and the realistic trapezoidal gradient pulses. Even though there are some differences (e.g. in the gradient amplitude, third row), the

approximately linear behavior of M_1 and M_2 is nearly equal. Dashed vertical lines denote different values of t_S (0 ms, 0.45 ms, 0.95 ms), the blueish region in the first row denotes the interval shown enlarged in the second row.
